# The Rapid Emergence of Ceftazidime-Avibactam Resistance Mediated by KPC Variants in Carbapenem-Resistant *Klebsiella pneumoniae* in Zhejiang Province, China

**DOI:** 10.3390/antibiotics11060731

**Published:** 2022-05-30

**Authors:** Congcong Liu, Yuchen Wu, Ling Huang, Yanyan Zhang, Qiaoling Sun, Jiayue Lu, Yu Zeng, Ning Dong, Chang Cai, Zhangqi Shen, Gongxiang Chen, Rong Zhang

**Affiliations:** 1 Department of Clinical Laboratory, School of Medicine, Second Affiliated Hospital of Zhejiang University, Hangzhou 310000, China; congliu@zju.edu.cn (C.L.); 22018229@zju.edu.cn (Y.W.); hl17788591051@163.com (L.H.); 12018247@zju.edu.cn (Y.Z.); sunqiaoling@zju.edu.cn (Q.S.); 12018248@zju.edu.cn (J.L.); 21818182@zju.edu.cn (Y.Z.); 2 Department of Clinical Laboratory Medicine, The Women’s and Children’s Hospital of Linping District, Hangzhou 310000, China; 3 Department of Medical Microbiology, School of Biology and Basic Medical Science, Medical College of Soochow University, Suzhou 215000, China; dongning@suda.edu.cn; 4 College of Animal Science and Technology, College of Veterinary Medicine, Zhejiang Agricultural and Forestry University, Hangzhou 310000, China; c.cai@Murdoch.edu.au; 5 Beijing Advanced Innovation Center for Food Nutrition and Human Health, College of Veterinary Medicine, China Agricultural University, Beijing 100000, China; szq@cau.edu.cn

**Keywords:** ceftazidime-avibactam, carbapenem-resistant *Klebsiella pneumoniae*, KPC variants, China

## Abstract

Ceftazidime-avibactam (CAV) is a new treatment option against carbapenem-resistant *Klebsiella pneumoniae* (CRKP) infections. However, the rapid emergence of CAV resistance mediated by KPC variants has posed a severe threat to healthcare after its clinical application. The characteristics of CAV resistance in CRKP strains needs to be determined in China. A total of 477 CRKP isolates were collected from 46 hospitals in Zhejiang Province from 2018 to 2021. The results demonstrated that CAV had a potent activity against 94.5% of all CRKP (451/477, 95% CI: 93.0–96.1%) and 86.0% of CRKP strains carrying *bla*_KPC_ genes (410/477, 95% CI: 83.5–88.4%). A total of 26 CAV-resistant strains were found. Among these strains, sixteen harbored metallo-β lactamases, and two carried KPC-2 carbapenemase and mutated *ompK35* and *ompK36*. Eight CRKP strains encoded KPC-33 or KPC-93, belonging to ST11, among which seven strains were detected in patients hospitalized in 2021 after exposure to CAV and one strain was associated with intra-hospital spread. CAV is a potent agent in vitro against CRKP strains. The rapid development of CAV resistance mediated by various KPC variants after a short period of CAV treatment has increased and brought difficulties in treating infections caused by CRKP strains, especially those belonging to ST11. The surveillance of bacterial resistance against CAV is highly recommended due to the steep development of CAV resistance and rapid evolution of KPC enzymes.

## 1. Introduction

The global spread of carbapenem-resistant *Klebsiella pneumoniae* (CRKP) is posing a serious threat to public health worldwide [1]. Previous studies have revealed that the prevalence of CRKP infection has increased noticeably in recent decades, and the mortality rate of bloodstream infection caused by CRKP strains has reached more than 70.0% [2,3]. Tigecycline and colistin are considered “last-resort antibiotics” against CRKP infections [4,5]. However, a number of reports have highlighted that the resistance to tigecycline [6,7] and colistin [8] is increasingly on the rise from mobile tigecycline resistance determinants, e.g., *tmexCD1-toprJ1*, and flavin-dependent mono-oxygenase *tet*(X) variants, and mobile colistin resistance determinants, such as *mcr* genes [8,9,10]. In addition, colistin treatment can cause neurotoxicity and nephrotoxicity [11].

A novel β-lactam/β-lactamase inhibitor combination called Ceftazidime-avibactam (CAV) is considered as one of the alternative therapies to treat CRKP infections. It has potent activity against CRKP with β-lactamases and extended-spectrum β-lactamases (ESBLs), Klebsiella pneumoniae carbapenemases (KPCs), AmpC, and certain oxacillinases (OXA), but not metallo-β lactamases, owing to the new non-β-lactam/β-lactamase inhibitor avibactam. It protects ceftazidime from degradation and reduces the minimum inhibitory concentration (MIC) of ceftazidime against Enterobacteriaceae producing certain β-lactamases. Therefore, CAV reverses the resistance to ceftazidime induced by ESBLs and AmpC via interacting with the active-site serine of these enzymes to form a covalent adduct reversibly [12]. A large multicenter cohort study found that CAV treatment was the sole independent predictor of survival in patients with KPC-producing *K. pneumoniae* (KPC-KP) bacteremia and concluded that CAV was a potential antimicrobial agent against severe KPC-KP infections [13].

As an efficacy treatment against KPC carbapenemase production, CAV was approved for the clinical treatment of complicated intra-abdominal and urinary tract infections in the USA in 2015 and was allowed in China in September 2019 [14,15]. Recently, significant increases in CAV resistance in KPC-KP strains have been observed. Mutations within the Ω-loop of KPC-2/KPC-3 enzymes were the principal resistance mechanisms observed in KPC-KP strains apart from the co-production of metallo-β lactamases [15,16,17]. The increased expression of KPC carbapenemases with porin deficiency was also related to CAV resistance in *K. pneumoniae* strains producing KPC-2/KPC-3 enzymes [18,19,20,21,22]. To evaluate the efficacy of this newly approved therapy and investigate the development of its resistance after two years of clinical application in Zhejiang Province, China, we collected 477 CRKP isolates from 2018 to 2021 to test the in vitro activity of CAV, and analyzed the mechanisms underlying CAV resistance in KPC-KP strains.

## 2. Results

### 2.1. Carbapenem-Resistant K. pneumoniae Isolates

**Figure 1:**
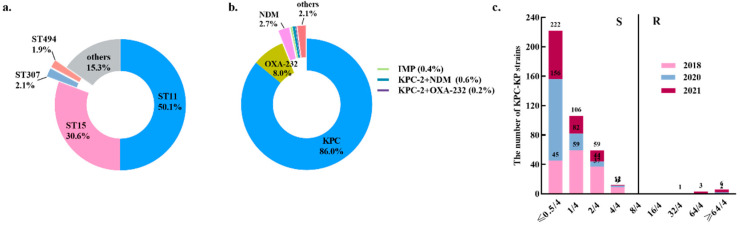
The distribution of carbapenemases, MLST and MIC values of CAV among 477 CRKP strains. (**a**) The distribution of carbapenemases. (**b**) The distribution of MLST. (**c**) The comparison of MIC values of CAV in 477 CRKP strains collected during 2018–2021.

A total of 477 non-duplicated CRKP strains were from Zhejiang Province, China, during 2018–2021. ST11 was the most prevalent sequence type (ST) (50.1%, 239/477, 95% CI: 46.6–53.6%), followed by ST15 (30.6%, 146/477, 95% CI: 27.4–33.8%) (Figure 1a). A total of 467 CRKP isolates carried the carbapenemase genes, including *bla*_KPC_ (n = 410, 86.0%, 95% CI: 83.5–88.4%), *bla*_OXA-232_ (n = 38, 8.0%, 95% CI: 6.1–9.8%), *bla*_NDM_ (n = 13, 2.7%, 95% CI: 1.6–3.9%), and *bla*_IMP_ (n = 2, 0.4%, 95% CI: 0.1–1.5%). The co-occurrence of *bla*_KPC-2_ and *bla*_NDM_ was detected in three strains, accounting for 0.6% (n = 3, 95% CI: 0.1–1.8%) and one strain (n = 1, 0.2%, 95% CI: 0.1–1.2%) carried both *bla*_KPC_ and *bla*_OXA-232_. The KPC enzyme was the most frequently detected carbapenemase in these strains (86.0%, 410/477) (Figure 1b). The high-resistance proportions of ceftazidime, cefotaxime, cefepime, piperacillin/tazobactam, cefoperazone/sulbactam, ciprofloxacin and aztreonam among these CRKP strains were 99.2% (95% CI: 97.9–99.8%), 99.2% (95% CI: 97.9–99.8%), 97.1% (95% CI: 95.9–98.2%), 99.0% (95% CI: 97.6–99.7%), 97.9% (95% CI: 96.9–98.9%), 90.8% (95% CI: 88.8–92.8%) and 98.3% (95% CI: 97.4–99.2%), respectively. In addition, the CRKP strains manifested resistance to cefmetazole at 66.0% (95% CI: 62.8–69.3%) and amikacin at 50.3% (95% CI: 46.8–53.8%) (Table 1 and Table S1).

### 2.2. Resistance Profile of the CRKP Strains to Ceftazidime-Avibactam

**Figure 2:**
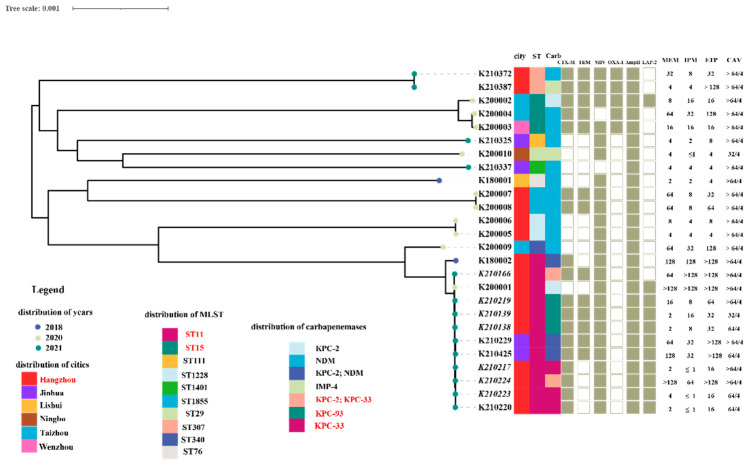
The overview of microbiological and molecular characteristics of 26 CAV-resistant CRKP isolates. It consisted of phylogenetic analysis, distribution of separation time, location, MLST, carbapenemases, the other β-lactamases and the antimicrobial susceptibility of carbapenems and CAV. The names of those strains whose patients were treated with CAV are shown in italics.

MIC of CAV against 477 CRKP strains ranged from ≤0.5/4 to >64/4 μg/mL and MIC_50_ and MIC_90_ were ≤0.5/4 μg/mL and 2/4 μg/mL, respectively (Table 1; Figure 1c). A total of 26 strains (5.5%, 95% CI: 3.9–7.0%) were resistant to CAV (MIC ≥ 16/4 μg/mL) and were recovered from six administrative districts of Zhejiang Province, namely Hangzhou (n = 14, 53.8%, 95% CI: 50.4–57.3%), Jinhua (n = 4, 15.4%, 95% CI: 4.4–34.9%), Taizhou (n = 3, 11.5%, 95% CI: 2.4–30.2%), Wenzhou (n = 2, 7.7%, 95% CI: 0.9–25.1%), Ningbo (n = 2, 7.7%, 95% CI: 0.9–25.1%), and Lishui (n = 1, 3.8%, 95% CI: 0.1–19.6%) (Figure 2). Thirteen of the twenty-six CAV-resistant CRKP strains with high MICs (≥32/4 μg/mL) produced metallo-β lactamases, including NDM (n = 11, 84.6%) and IMP (n = 2, 15.4%). Co-production of NDM and KPC-2 carbapenemases was detected in three CRKP strains with CAV resistance isolated during 2018–2021 and all other CAV-resistant strains harbored the *bla*_KPC_ gene. In total, 94.5% of CRKP strains (451/477, CI: 93.0–96.1%) were susceptible to CAV, including all OXA-232-producing CRKP strains (n = 38) and 400 KPC-2-producing strains.

Among the ten KPC-producing *K. pneumoniae* isolates, two carried wild-type KPC-2 β-lactamases solely, three carried *bla*_KPC-93_ and the remanent of CRKP isolates were encoded KPC-33. Among these, two CRKP strains possessed both KPC-2 and KPC-33 enzymes. All CRKP strains were recovered from patients who were treated with CAV after 23–41 days, except one, which was related to nosocomial transmission. Nine out of ten strains consistently had the ST11 clonal background, with the remaining one, numbered K200002, belonging to the ST15 clone. They were clustered into the same clade based on phylogenetic analysis. In contrast, CAV-resistant CRKP isolates producing metallo-β lactamases, including those encoding both KPC-2 and NDM carbapenemases, were genetically diverse and belonged to ST11 (n = 3), ST15 (n = 2), ST1228 (n = 2), ST1855 (n = 2), ST307 (n = 2), ST76 (n = 1), ST340 (n = 1), ST29 (n = 1), ST111 (n = 1) and ST1401 (n = 1), respectively. The phylogenetic analysis revealed that they were grouped into ten distinct clades (Figure 2).

### 2.3. Mechanisms of CAV Resistance in KPC-KP Strains

The KPC-33 carbapenemase carried a substitution of the aspartic acid residue at amino acid positions 179 with tyrosine (D179Y) in the Ω loop of KPC carbapenemases and were probably derived from KPC-2. This mutation impaired its capacity to hydrolyze carbapenems but enhanced the ability to resist CAV. Three KPC-33-positive strains with high MIC values of ≥64 μg/mL for CAV were susceptible to imipenem and showed intermediate susceptibility or low-level resistance to meropenem and ertapenem (meropenem MIC range from 2 to 4 µg/mL; imipenem MIC ≤ 1 µg/mL; ertapenem MIC = 16 µg/mL) (Figure 2). The co-production of KPC-2 carbapenemase in KPC-33-positive strains enabled them to highly resist carbapenems and retained a high level of resistance to CAV simultaneously (meropenem, imipenem and ertapenem, MIC ≥ 64 µg/mL) (Figure 2).

The novel KPC variant, KPC-93, shared 98.3% amino acid sequence homology with KPC-2 carbapenemases and contained a five-amino-acid insertion (Asn-Arg-Ala-pro-Asn) located between amino acids 266 and 267 compared to KPC-2, of which, the sequence from amino acids 263 to 266 was duplicated with the insertion fragment Arg-Ala-pro-Asn in KPC-93.

To understand the resistant mechanism of the two strains producing wild-type KPC-2 carbapenemases, the porin deficiency and mutations of the porins were investigated. Mutaions in *ompK35* and *ompK36* in these strains were observed, each exhibiting 99.2%~99.4% and 91.9%~99.5% nucleotide sequence similarity with wild-type *ompK35* and *ompK36*, respectively. In addition, amino acid changes have occurred in these two strains for the two porins.

## 3. Discussion

CAV was recently approved for the treatment of KPC-KP infections around the world and in China [3,15]. However, since the application of CAV in the clinic, there has been an increasing number of reports regarding CAV resistance [15,16,17,23]. In the current study, we investigated the characteristics of CAV resistance in 477 CRKP strains in Zhejiang Province, China, during 2018–2021, including the marketing time of CAV. Our data indicate that the exposure to CAV led to the rapid evolution of KPC variants, which have become the principal mechanism underlying CAV resistance in Zhejiang Province, China.

In general, the prevalence of CAV resistance was low in CRKP strains in China. The production of metallo-β-lactamases and increased expression of the wild-type KPC-2 carbapenemases in combination with porin deficiency were the predominant mechanisms of CAV resistance before it entered the Chinese market [23,24,25]. Similarly, only two strains conferred resistance to CAV via merely carrying NDM carbapenemases and co-possessing NDM and KPC-2 carbapenemases, respectively. The CAV-resistant CRKP prevalence rate of 1.2% (95% CI: 0.1–4.2%) in our study was slightly lower than that (3.7%) in another study from China [24], indicating that CAV was indeed a potent potential substitute for carbapenems in China, as expected before the introduction into the Chinese market. However, the rapid emergence of CAV resistance in CRKP strains after exposure to CAV was observed, hinting that the application of CAV has accelerated the dispersal of CAV-resistant CRKP strains in China. Among the CAV-resistant CRKP strains identified, 53.8% were distributed in Hangzhou, probably attributing partly to the fact that CAV had been more frequently prescribed in this capital city of Zhejiang, with more tertiary care hospitals, and that a very high percentage of CRKP strains (40.0%, 191/477, 95% CI: 36.6–43.4%) were collected from Hangzhou.

Recent studies have indicated that the mutations on KPC-2 and KPC-3 carbapenemases have emerged as another primary mechanism mediating CAV resistance in KPC-KP strains. A large number of KPC variants have sprung up nearly two decades after the discovery of KPC-2 in 1996 due to the clinical application of CAV [17,23,24,26,27]. To date, 108 isoforms of KPC carbapenemases have been registered on the NCBI web site [28]. It was reported that the variations contributing to CAV resistance mainly occurred in the KPC Ω-loop region composed of 164Arg-179Asp, encircling the core of the active site of KPC carbapenemases in substrates acylation and deacylation, including KPC-31 and KPC-33, KPC-35, KPC-51, KPC-52 and KPC-57 variants [15,16,17,23,29,30,31]. The most common D179Y mutation in KPC-2 and KPC-3 enzymes reduced the inhibitory effect of avibactam and maintained ceftazidime-hydrolyzing activity, giving rise to KPC-31 and the most common KPC-33 variants in China, respectively [15,16]. So far, KPC-33-producing and CAV-resistant CRKP strains have already appeared in Shanghai, Zhejiang and Henan provinces and they tended to present low-level carbapenem resistance [15,24,32]. Shi et al. reported that the selective pressure from CAV usage resulted in the transformation of KPC-2 to KPC-33 carbapenemase, contributing to the reduced resistance to carbapenems but enhanced resistance to CAV in these strains [15]. Likewise, a total of seven strains carrying KPC variants were isolated from patients with a history of CAV usage. Three CRKP strains producing only KPC-33 in our study exhibited similar resistance profiles of carbapenem and CAV with Shi’s reports [15]. Two of them, K210217 and K210223, were isolated from patients after exposure to this drug for 23 and 41 days, respectively. Another KPC-33-positive strain, K210220, was obtained from a patient in the same hospital without a history of CAV use and the clonal relatedness was detected in these isolates with an ST11 clone background, indicating the potential for the horizontal transmission of KPC-33 carbapenemase within the hospital in a small range. Of note, the incorporation of the KPC-2 enzyme into KPC-33-producing *K. pneumoniae* strains, for instance, K210224 and K210166, might enhance the phenotype of carbapenem resistance, similar to previous reports [23,24], making the anti-infection treatment even worse.

The novel variant, KPC-93, with a five-amino-acid insertion (Asn-Arg-Ala-pro-Asn), was identified in our study. It mediated the modest resistance to carbapenem and high-level resistance to CAV in ST11 CRKP strains. The mutations happened in the vicinity regions of the Ω-loop and the hinge loop, consisting of amino acids 263–277. There has been another “hotspot” region of mutation (amino acids 240–243) leading to CAV resistance since 2015. This region was close to the hinge loop encompassing the active site of KPC and participated in the CAV resistance mediated by the *bla*_KPC-14_ and *bla*_KPC-28_ genes [27,33]. Thus, the KPC β-lactamases were prone to developing diverse mutations under the selection pressure of CAV, indicating that pertinent utilization and sustained surveillance were essential to prevent the emergence of novel KPC variants conferring both high resistance to CAV and high resistance to carbapenems. The ST11 clone, which often presented as the multi-drug resistance phenotype and was highly transmissible, has been reported to be the dominant epidemic genetic lineage among CRKP strains in China [34]. CAV resistance encoded by *bla*_KPC_ variants has merely emerged in ST11-type CRKP strains in China [15,23,24,32] and all eight CRKP strains carrying KPC variants in the present study also belonged to the ST11 clone, indicating that this pandemic clone was the major reservoir of KPC variants and CAV resistance in China.

Herein, two CRKP isolates (K200001 and K20002) encoded the wild-type KPC-2 carbapenemase but exhibited resistance to CAV (MIC, >64/4 μg/mL). Though mutations of OmpK35 and OmpK36 were identified from these strains, it is unclear whether the mutated OmpK35 and OmpK36 contribute to their resistance to CAV as reported in other studies [21,22], which needs to be further investigated.

Although it appears that CAV is a promising antibiotic for KPC-KP infection, what is not negligible is that CAV is ineffective against metallo-β lactamase-producing strains, though the therapeutic regime of aztreonam and CAV combination might work [35,36]. In addition, one study reported that 23.0% of CRE infections recurred within 90 days after successful treatment by CAV, some even as few as 10 days [37]. So, it is still necessary to discover new inhibitors and combination antimicrobial regimens that are active against CRKP infections.

There are some limitations in this study. First, we were unable to obtain information as to whether the collected CRKP strains were from infections or colonizations for some patients due to the lack of their informed consent. Second, we do not have access to strains and data in 2019.

## 4. Materials and Methods

### 4.1. Sample Collection

Clinical CRKP strains were collected from 46 hospitals in 11 cities in Zhejiang Province, China, from 2018 to 2021. All isolates were identified via matrix-assisted laser desorption ionization–time of flight mass spectrometer (MALDI-TOF MS) (Bruker Daltonik GmbH, Bremen, Germany) and 16S rRNA gene sequencing.

### 4.2. Antimicrobial Susceptibility Testing and Carbapenemase-Encoding Gene Screening

Antimicrobial susceptibility testing was conducted using a broth microdilution method to assess the MIC values of 15 antibiotics (imipenem, meropenem, ertapenem, ceftazidime, cefotaxime, cefmetazole, cefepime, aztreonam, amikacin, ciprofloxacin, colistin, tigecycline, piperacillin/tazobactam, cefoperazone/sulbactam and ceftazidime/avibactam) against carbapenem-resistant *K. pneumoniae* isolates. The results were interpreted according to the Clinical and Laboratory Standards Institute guideline, except for tigecycline, which was interpreted based on the European Committee on Antimicrobial Susceptibility Testing (EUCAST) breakpoints [38,39]. Both PCR and Sanger sequencing were performed to screen the carbapenemase-encoding genes *bla*_NDM_, *bla*_IMP_, *bla*_KPC_, *bla*_OXA-48_ and *bla*_VIM_, including *bla*_KPC_ variants, as described previously [40].

### 4.3. Whole Genome Sequencing and Bioinformatics Analysis

Genomic DNA was extracted using a genomic DNA extraction kit (TIANGEN^®^, Beijing, China) and sequenced using the Illumina Hiseq 2500 platform with a 2 × 150 bp paired-end sequencing strategy [41]. De novo genome assembly was performed using SPAdes Genome Assembler version 3.11.1 (created by Anton Bankevich, et al., St. Petersburg, Russia) [42]. The annotation of assembled genome sequences was generated by the RAST tool and modified manually (created by Ross Overbeek, et al., Burr Ridge, IL, USA) [43]. Multi-locus sequence typing, antimicrobial resistance gene and virulence gene screening were conducted with Kleborate version 2.0.4 (created by Kathryn E Holt, Melbourne, Victoria, Australia) [44]. Plasmid replicons were analyzed using PlasmidFinder version 2.1 (created by Alessandra Carattoli, et al., Rome, Italy) [45]. The core genome phylogenetic tree of all CAV-resistant CRKP strains was generated using the Harvest suite (created by Todd J Treangen, et al., e, Frederick, MD, USA) [46]. The phylogenetic tree was visualized and modified using iTOL version 4 (created by Ivica Letunic and Peer Bork, Heidelberg, Germany) [47]. The wild-type *ompK35* (accession number KX528047.1) and *ompK36* (accession number KY086540.1) nucleotide sequences were used as the reference sequences to compare the relevant sequences of *ompK35* and *ompK36* of the CAV-resistant and wild-type KPC producing strains.

### 4.4. Statistical Analysis

The 95% confidence intervals (CI) were calculated using the approximate normal distribution method or exact probabilities method (binom.test in R), as appropriate.

## 5. Conclusions

In conclusion, though CAV exhibited a potent efficacy against most CRKP strains in our study, the rapid emergence of CAV resistance mediated by KPC variants among CRKP strains after exposure to CAV has brought new challenges to clinical treatment. The surveillance of CAV resistance and rational therapeutic regimens is highly suggested for clinicians to maximize the efficacy of CAV.

## Figures and Tables

**Table 1:** Susceptibility of 477 CRKP strains to commonly used antibiotics.

Antibiotic	MIC_50_ (μg/m)	MIC_90_ (μg/mL)	Range (μg/mL)	R%	I%	S%
Imipenem	32	64	≤1 –> 128	90.1%	3.4%	6.5%
Meropenem	64	128	≤1 –> 128	93.5%	3.8%	2.7%
Ertapenem	128	>128	≤0.5 –> 128	99.6%	0.0%	0.4%
Cefmetazole	128	>128	≤2 –> 128	66.0%	9.2%	24.7%
Ceftazidime	128	>128	≤2 –> 128	99.2%	0.4%	0.4%
Cefotaxime	>128	>128	≤1 –> 128	99.2%	0.0%	0.8%
Piperacillin/Tazobactam	>256/4	>256/4	16/4 –> 256/4	99.0%	0.8%	0.2%
Cefoperazone/Sulbactam	256/128	>256/128	32/16 –> 256/128	97.9%	2.1%	0.0%
Ceftazidime/avibactam	≤0.5/4	2/4	≤0.5/4 –> 64/4	5.5%	-	94.5%
Cefepime	>64	>64	≤2 –> 64	97.1%	-	1.3%
Colistin	≤0.5	1	≤0.5 –> 8	1.5%	98.5%	-
Tigecycline	0.5	2	≤0.25 –> 8	30.2%	-	69.8%
Ciprofloxacin	>32	>32	≤0.25 –> 32	90.8%	0.0%	9.2%
Amikacin	128	>128	≤4 –> 128	50.3%	0.4%	49.3%
Aztreonam	>128	>128	≤4 –> 128	98.3%	0.4%	1.3%

## Data Availability

The draft genome sequences of all CAV-resistant CRKP strains were deposited in the GenBank database under the BioProject number: PRJNA774095.

## Supplementary Materials

The following supporting information can be downloaded at: https://www.mdpi.com/article/10.3390/antibiotics11060731/s1, Table S1: Antibiotic susceptibility profiles of 26 CAV-resistant strains in this study.
