# Supplementary material for: The Rapid Emergence of Ceftazidime-Avibactam Resistance Mediated by KPC Variants in Carbapenem-Resistant Klebsiella pneumoniae in Zhejiang Province, China
Source: Antibiotics (Basel). 2022 May 30;11(6):731. doi: 10.3390/antibiotics11060731 (PMC9219983; doi:10.3390/antibiotics11060731)
Supplement: Supplementary file 1 [file antibiotics-11-00731-s001.zip › antibiotics-1671053-supplementary.pdf]

**Supplementary**

**Table S1. Antibiotic susceptibility profiles of 26 CAV-resistant strains in this study.**

| strain | IPM | MEM | ETP | CMZ | CAZ | CTX | TZP | SCF | CAV | FEP | CO | TGC | CIP | AK | ATM |
|---|---|---|---|---|---|---|---|---|---|---|---|---|---|---|---|
| K180001 | 2 | 2 | 4 | 8 | >128 | 128 | 256/4 | 128/64 | >64/4 | 16 | 1 | ≤0.25 | ≤1 | ≤4 | >128 |
| K180002 | 128 | 128 | >128 | >128 | >128 | >128 | >256/4 | >256/128 | >64/4 | >64 | ≤0.5 | ≤0.25 | >32 | ≤4 | >128 |
| K200001 | >128 | >128 | >128 | >128 | >128 | >128 | >256/4 | >256/128 | >64/4 | >64 | ≤0.5 | ≤0.25 | >32 | ≤4 | >128 |
| K200002 | 8 | 16 | 16 | 64 | >128 | >128 | >256/4 | >256/128 | >64/4 | >64 | ≤0.5 | 0.5 | >32 | ≤4 | >128 |
| K200003 | 16 | 16 | 16 | 32 | >128 | >128 | >256/4 | >256/128 | >64/4 | 64 | ≤0.5 | ≤0.25 | 32 | ≤4 | >128 |
| K200004 | 32 | 64 | 128 | >128 | >128 | >128 | >256/4 | >256/128 | >64/4 | >64 | ≤0.5 | ≤0.25 | >32 | ≤4 | >128 |
| K200005 | 4 | 4 | 4 | 8 | >128 | 64 | 128/4 | 128/64 | >64/4 | 16 | 1 | ≤0.25 | ≤1 | ≤4 | ≤4 |
| K200006 | 4 | 8 | 8 | 8 | >128 | 64 | 128/4 | 128/64 | >64/4 | 8 | ≤0.5 | ≤0.25 | ≤1 | ≤4 | ≤4 |
| K200007 | 8 | 64 | 32 | 64 | >128 | >128 | >256/4 | >256/128 | >64/4 | >64 | 1 | ≤0.25 | 16 | ≤4 | 32 |
| K200008 | 8 | 64 | 64 | 64 | >128 | >128 | >256/4 | >256/128 | >64/4 | >64 | ≤0.5 | ≤0.25 | 16 | ≤4 | 32 |
| K200009 | 32 | 64 | 128 | >128 | >128 | >128 | >256/4 | >256/128 | >64/4 | >64 | ≤0.5 | ≤0.25 | >32 | ≤4 | 8 |
| K200010 | ⩽1 | 4 | 4 | >128 | >128 | 128 | >256/4 | 128/64 | 32/4 | 64 | 1 | 2 | 4 | 128 | >128 |
| K210138 | 8 | 2 | 32 | 64 | >128 | >128 | >256/4 | 256/128 | 64/4 | >64 | 1 | 1 | >32 | >128 | >128 |
| K210139 | 16 | 2 | 32 | 64 | >128 | >128 | >256/4 | 256/128 | 32/4 | >64 | 1 | 1 | >32 | >128 | >128 |
| K210166 | >128 | 64 | >128 | >128 | >128 | >128 | >256/4 | >256/128 | >64/4 | >64 | ≤0.5 | 1 | >32 | >128 | >128 |
| K210217 | ⩽1 | 2 | 16 | 128 | >128 | >128 | >256/4 | 256/128 | >64/4 | >64 | ≤0.5 | 4 | >32 | 128 | >128 |
| K210219 | 8 | 16 | 64 | 64 | >128 | >128 | >256/4 | 256/128 | >64/4 | >64 | ≤0.5 | 1 | >32 | >128 | >128 |
| K210220 | ⩽1 | 2 | 16 | 64 | >128 | >128 | >256/4 | 256/128 | 64/4 | >64 | ≤0.5 | 2 | >32 | 128 | >128 |
| K210223 | ⩽1 | 4 | 16 | 64 | >128 | >128 | >256/4 | 256/128 | 64/4 | >64 | 2 | 4 | >32 | 64 | >128 |
| K210224 | 64 | >128 | >128 | >128 | >128 | >128 | >256/4 | >256/128 | >64/4 | >64 | ≤0.5 | 2 | >32 | 128 | >128 |
| K210229 | 32 | 64 | >128 | >128 | >128 | >128 | >256/4 | >256/128 | >64/4 | >64 | 1 | 2 | >32 | >128 | >128 |
| K210325 | 2 | 4 | 8 | 8 | >128 | 128 | 128/4 | 128/64 | >64/4 | 16 | 1 | 0.5 | ≤1 | ≤4 | ≤4 |
| K210337 | 4 | 4 | 4 | 8 | >128 | 128 | 256/4 | 128/64 | >64/4 | 16 | 1 | ≤0.25 | ≤1 | ≤4 | 64 |

| | | | | | | | | | | | | | | | |
|---|---|---|---|---|---|---|---|---|---|---|---|---|---|---|---|
| K210372 | 8 | 32 | 32 | 32 | >128 | >128 | >256/4 | >256/128 | >64/4 | >64 | 1 | 0.5 | 32 | ≤4 | 64 |
| K210387 | 4 | 4 | >128 | >128 | >128 | >128 | 64/4 | 128/64 | >64/4 | 32 | ≤0.5 | 0.5 | >32 | ≤4 | 32 |
| K210425 | 32 | 128 | >128 | >128 | >128 | >128 | >256/4 | >256/128 | 64/4 | >64 | 1 | 1 | >32 | >128 | >128 |

a IMP, imipenem; MEM, meropenem; ETP, ertapenem; CMZ, cefmetazole; CAZ: Ceftazidime; CTX: Cefotaxime; TZP: Piperacillin/Tazobactam; SCF: Cefoperazone/Sulbactam; CAV, ceftazidime/avibactam; FEP: Cefepime; CO: colistin; TGC, tigecycline; CIP, ciprofloxacin; AK, amikacin; ATM, aztreonam
